# Short‐Term Sleep Disruption Does Not Modify Responses to Experimentally Induced Knee Pain in Healthy Individuals

**DOI:** 10.1002/ejp.70260

**Published:** 2026-03-29

**Authors:** Emma Hertel, Aske Hostrup Graversen, Anders Bobo Larsen, Diego Martinez Echevarria, Michael Skipper Andersen, Lars Arendt‐Nielsen, Kristian Kjær‐Staal Petersen

**Affiliations:** ^1^ Department of Health Science and Technology, Faculty of Medicine Aalborg University Aalborg Denmark; ^2^ Center for Mathematical Modeling of Knee Osteoarthritis (MathKOA) Aalborg University Aalborg Denmark; ^3^ Center for Neuroplasticity and Pain (CNAP) Aalborg University Aalborg Denmark; ^4^ Department of Materials and Production, Faculty of Engineering and Science Aalborg University Aalborg Denmark; ^5^ Department of Gastroenterology and Hepatology, Mech‐Sense, Clinical Institute Aalborg University Hospital Aalborg Denmark; ^6^ Steno Diabetes Center North Denmark, Clinical Institute Aalborg University Hospital Aalborg Denmark

**Keywords:** biomechanics, forced awakenings, gait analysis, pain modulation, psychological vulnerability, quantitative sensory testing

## Abstract

**Background:**

Knee osteoarthritis and poor sleep quality often co‐occur and have a reciprocal relationship. Long‐term exposure to poor sleep can facilitate pain, and short‐lasting experimental sleep disturbances increase pain sensitivity. This study combined experimental knee pain and sleep disturbances to investigate whether short‐term sleep disruption increases experimental knee pain intensity and if baseline traits affect this vulnerability.

**Methods:**

Knee pain was induced using hypertonic saline (7%) injected into the infrapatellar fat pad (IFP) before and after three forced awakenings per night for three nights. Gait was measured using marker‐less motion capture. Pain sensitivity was assessed as pressure pain and tolerance thresholds, temporal summation of pain (TSP), and conditioned pain modulation. Questionnaires assessed sleep quality, pain catastrophizing, anxiety, and depression.

**Results:**

Thirty healthy participants experienced moderate‐to‐severe knee pain from the hypertonic saline injected into the IFP. Sleep disruption significantly reduced sleep quality. Exploratory analysis revealed that participants with baseline knee pain < 7 (VAS 0–10) experienced increased pain after the sleep disruption (*p* < 0.05). There were no changes in other measurements.

**Conclusions:**

This study investigated a potential novel pain model combining experimental knee pain and short‐term sleep disturbance but did not find any changes in knee pain intensity, pain sensitivity, psychological factors, or gait characteristics.

**Significance Statement:**

This study introduced an experimental model integrating sleep disruption and knee pain to probe individual pain vulnerability. Results revealed that short‐term sleep loss increased knee pain in individuals with lower baseline experimental knee pain intensity, and that a combination of psychophysical factors explained 30%–45% of the substantial variability in knee pain. Temporal summation emerged as a consistent predictor of knee pain, while sleep quality predicted pain after sleep disruption. These findings advance understanding of how sleep modulates individual pain vulnerability.

## 1 Introduction

Knee osteoarthritis (OA) has an estimated 527 million cases (Mora et al. 2018) and is associated with pain, structural changes, and altered biomechanics (Boyer and Hafer 2019; Neogi 2013). Pain and functional impairments are often the cause for patients to seek medical attention (Neogi 2013), and the severity of knee OA is determined based on the degree of cartilage degeneration (Kellgren and Lawrence 1957). However, the degree of cartilage degeneration has limited correlation with pain intensity (Felson 2005). Instead, knee OA is considered a multifactorial disease, and several factors have been linked to disease burden, including inflammation, cognitive factors, sleep, biomechanics of walking, and sensitivity of the nociceptive system (Boyer and Hafer 2019; Felson 2005; Hertel, Arendt‐Nielsen, et al. 2024; Li et al. 2020).

Quantitative sensory testing (QST) provides surrogate measurements of sensitivity changes in the central nervous system, which are known to influence outcomes in patients with knee OA (Petersen et al. 2023) and pain intensity in experimental pain models (McPhee and Graven‐Nielsen 2019). Motion capture can be used to quantify the biomechanical properties of walking, which are associated with vulnerability to movement‐evoked pain (Boyer and Hafer 2019) and pain intensity fluctuations in patients with knee OA (Maly et al. 2008). An estimated three‐quarters of patients with chronic pain conditions report sleep problems (Sun et al. 2021). Insufficient sleep in a short period of time can lead to pain and emotional manifestations such as stress, anxiety, and depression (Medic et al. 2017). Multiple biological mechanisms are shared between sleep and pain, and thus, disruption of one will eventually affect the other, enabling mutual exacerbation (Finan et al. 2013). In patients with chronic pain, poor sleep quality often aggravates existing symptoms of pain (Wiklund et al. 2020). Further, poor sleep might also affect gait control, decreasing sensorimotor control (Umemura et al. 2021) and slowing walking speed (Kasović et al. 2021). Experimental models of pain can be utilized to study how individual variations in these factors might influence pain development.

The current study utilizes a novel experimental pain model combining hypertonic saline injections into the infrapatellar fat pad and repeated sleep disruptions to mimic features of knee OA. Hypertonic saline injections provoke pain along with alterations of gait (Bennell et al. 2004; Henriksen et al. 2011; Hodges et al. 2009), while sleep disruptions can increase pain sensitivity through increases in inflammatory mediators (Haack et al. 2020; Irwin et al. 2016), impaired emotional modulation (Huber et al. 2022), and impaired descending pain modulation (Staffe et al. 2019). The current work aimed to investigate whether experimentally reduced sleep quality might amplify the effects of experimental knee pain provocation and whether individual vulnerability to this can be predicted using psychological and physical baseline factors.

## 2 Methodology

### 2.1 Study Overview

**FIGURE 1:**
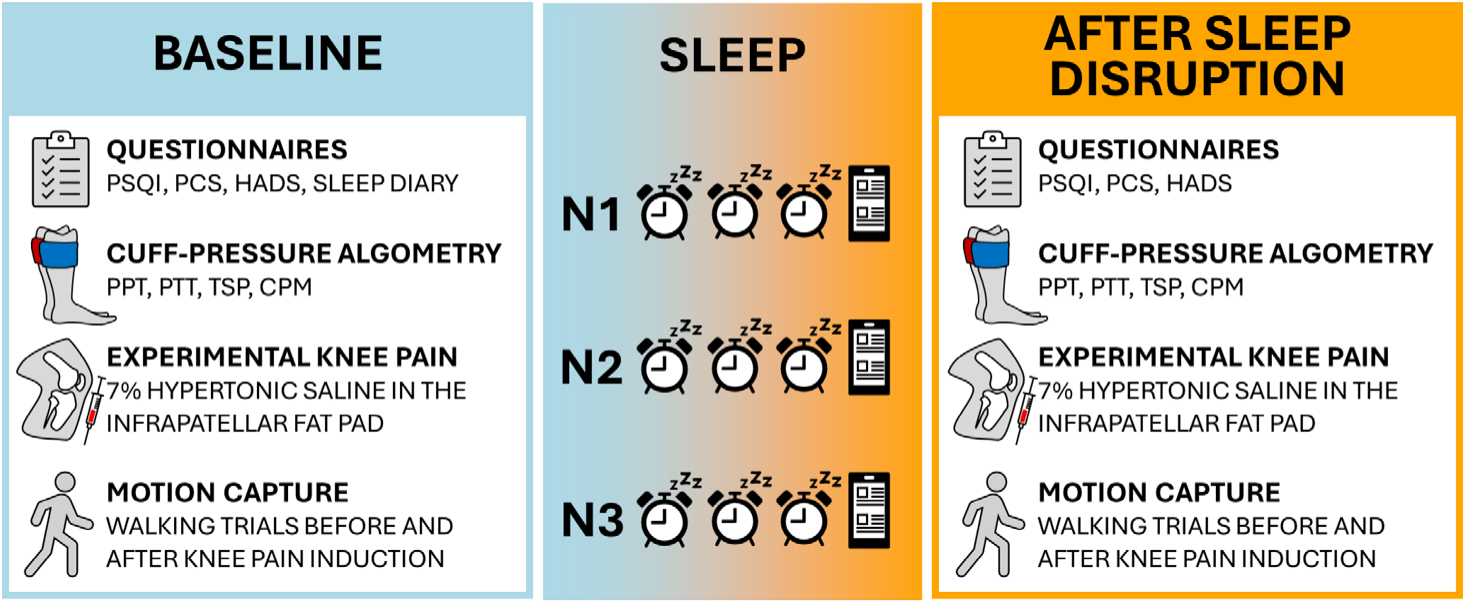
Overview of study design. HADS, hospital anxiety and depression scale; N, Night; PCS, the pain catastrophizing scale; CPM, conditioned pain modulation; PPT, pressure pain threshold; PSQI, Pittsburgh sleep quality index; PTT, pressure tolerance threshold; TSP, temporal summation of pain.

This study aimed to investigate the effects of disrupted sleep on experimental knee pain, pain sensitivity, and gait biomechanics in healthy volunteers. The primary research question was whether disrupted sleep increases experimental knee pain and the underlying mechanisms. Exploratory aims included whether baseline parameters could explain variability in peak knee pain intensities before and after experimental sleep disruption. See Figure 1 for an overview of the study protocol.

Volunteers attended two experimental sessions separated by three consecutive nights with experimentally disrupted sleep. During the experimental session, the volunteers answered a series of validated questionnaires, had their pain sensitivity assessed, and had their gait biomechanics captured in a pain‐free state. Sequentially, they received a painful saline injection in the infrapatellar fat pad (IFP). During the subsequent approximate 10‐min window of pain, they rated their pain every 30 s on an NRS (0 ‘no pain’ and 10 ‘worst pain’) and had their gait kinematics estimated during knee pain. Every morning during the study period, the volunteers completed a sleep diary to evaluate their quality of sleep. Ethical approval was granted by the North Denmark Region Committee on Health Research Ethics (N‐20220063) and the study was registered on ClinicalTrials.gov (NCT06336109).

### 2.2 Sample Size

No previous studies have investigated how experimental sleep disruption impacts experimentally induced hypertonic saline pain. Therefore, a medium‐to‐large effect size of sleep disruption on experimental pain was estimated (Cohen's d = 0.55). A study with a power of 80% and an alpha value of 0.05 should include at least 28 subjects. To account for dropouts, a total of 30 subjects were recruited.

### 2.3 Questionnaires

The Knee Injury and Osteoarthritis Outcome Score (KOOS) was used to estimate knee symptoms across the five subscales: pain (9 items), activities of daily living (ADL, 17 items), sport and recreation function (Sport/Rec, 5 items), knee‐related quality of life (QoL, 4 items), and other symptoms (7 items). The subscales are scored on a scale from no knee problems (score of 0) to extreme knee problems (score of 100). (Collins et al. 2016) The pain catastrophizing scale (PCS) was used to measure thoughts and feelings related to being in pain across 13 items. This is summarized as a single score ranging from zero to 52, with higher scores indicating an increased tendency to catastrophize during pain. (Sullivan et al. 1995) The Hospital Anxiety and Depression Scale (HADS) measures symptoms of anxiety and depression across two seven‐item subscales. Each subscale ranges from zero to 21, with higher scores indicating increased presence of symptoms. (Bjelland et al. 2002) Finally, the Pittsburgh Sleep Quality Index (PSQI) estimates quality of sleep across 19 items. This is summarized as a global score ranging from zero to 21, with higher scores denoting worse quality of sleep (Buysse et al. 1989).

### 2.4 Computer‐Controlled Cuff‐Pressure Algometry

A computer‐controlled cuff‐pressure algometer (Cortex Technology, Aalborg University), an electronic visual analogue scale (eVAS), and a pair of 13‐cm wide air cuffs were used to probe pain sensitivity. This system has excellent‐to‐good reliability for the tests used in the current study (Imai et al. 2016) and has previously been used for similar purposes (Hertel, Sathiyalingam, et al. 2024; Joensen et al. 2025; McPhee and Graven‐Nielsen 2019). The eVAS was sampled at 10 Hz and was anchored at 0 ‘no pain’ and 10 ‘worst pain imaginable’ and equipped with a stop button to terminate the stimulation. The air cuffs were placed on the widest part of the calves, and the dominant leg was determined as the index leg.

A ramped inflation at 1 kPa/s was administered on the index leg to determine the pressure pain threshold (PPT) and pressure tolerance threshold (PTT). The PPT was defined as the kPa pressure intensity when the eVAS reached one, and the PTT as the kPa pressure intensity when the stop button was pushed. The volunteer was instructed to push the stop button when the pressure became intolerable. To estimate temporal summation of pain (TSP), a train of 10 stimulations at the level of the PTT was administered on the index leg with one‐second stimulations and one‐second interstimulus intervals (Graven‐Nielsen et al. 2015; Petersen et al. 2016). The mean pain intensity of the last three inflations was subtracted from the mean of the first four inflations to calculate the TSP. To estimate conditioned pain modulation (CPM), a conditioning stimulus at 70% of the PTT was administered on the contralateral leg simultaneously with a ramped inflation at 1 kPa/s on the index leg. Participants were asked to rate the ramped inflation on the eVAS (Graven‐Nielsen et al. 2017; Imai et al. 2016; Petersen et al. 2019). The difference in PPT from the ramp with and without conditioning was determined as the conditioning pain modulation (CPM) effect.

### 2.5 Motion Capture

OpenCap [Stanford University, California] was used to capture three gait trials per condition (with and without pain before and after sleep disruption). The experimental setup consisted of two phones mounted on tripods at 30–45 degrees off the sagittal plane of the volunteer when walking to avoid segment occlusion and following OpenCaps' best‐practice recommendations (OpenCap, 2025). Before the gait trials, a standing reference was recorded with the volunteer in a neutral position. The volunteers then walked back and forth along a line without exiting the capture volume during the trial. The three trials per condition were recorded at random times, unknown to the volunteers, to capture the most natural gait pattern. Speed, stride length, and peak knee flexion were extracted for analysis. Speed and stride length were normalized by body height. Knee symmetry was calculated as x_dom/(x_dom + x_opp) based on previous definition (Enqvist et al. 2025). OpenCap has previously been demonstrated to accurately capture kinematics and predict dynamics of human movement (Svetek et al. 2025).

### 2.6 Experimental Knee Pain

A 0.25 mL bolus of 7% hypertonic saline was injected into the infrapatellar fat pad (IFP) at a 45‐degree angle in a superolateral direction in accordance with previous protocols (Bennell et al. 2004; Hodges et al. 2009). The IFP was palpated as the equidistant point of the patellar tendon and the condyles of the tibial and femoral bones. This has previously been demonstrated to produce knee pain and altered gait biomechanics, with the pain peaking after approximately 3 min, followed by a gradual decline over 15 min (Henriksen et al. 2011; Hodges et al. 2009). Importantly, hypertonic saline injections have previously been demonstrated to be suitable for repeated exposure with no evidence of relevant habituation (Smith et al. 2023). The knee pain intensity was rated on an NRS anchored at zero ‘no pain’ and 10 ‘worst pain’ every 30 s for 10 min starting immediately after the injection.

### 2.7 Experimental Sleep Disruption

The volunteers' sleep continuity was experimentally disrupted three times per night for three consecutive nights to simulate the fragmented sleep pattern and average number of awakenings reported by patients with chronic pain (Morin et al. 1998). Moreover, this protocol has previously been demonstrated to increase sensitivity to painful stimulation and change measures of pain sensitivity (Hertel et al. 2023; Joensen et al. 2025). During the first experimental session, alarms were scheduled on the volunteers' phones for the designated awake times (00:00, 02:30, and 05:00). To document each awakening, the volunteer was instructed to turn on the lights, capture a photo outside their bedroom, and immediately forward it to the research team. The timestamp on the messages, along with the pictures, was used as documentation. A maximum of two missed assignments was deemed acceptable. This protocol has previously been demonstrated to decrease quality of sleep and increase pain sensitivity (Joensen et al. 2025). Every morning during the experiment, the volunteer received a sleep diary with questions regarding quality of sleep (0 ‘worst quality’ to 100 ‘best quality’), level of rest (0 ‘no rest’ and 10 ‘best rest’), and sleep timings.

### 2.8 Statistics

All numeric scale data is presented as mean and standard deviation (SD) unless otherwise stated. Self‐reported sleep parameters (quality and level of rest) measured at baseline and after the three experimental nights were compared using repeated‐measures (RM) ANOVA. Biomechanical properties (speed, stride length, peak knee flexion, and knee symmetry) measured before and after knee pain induction at both baseline and after the sleep disruption were compared using RM‐ANOVA. The effects of minutes (after the injection) and day (before and after the sleep disruption) on knee pain intensity were investigated using RM‐ANOVA with two factors: day (two levels) and time (21 levels). When significant main effects were detected, a Bonferroni‐corrected post hoc analysis was conducted. Assumptions were checked using appropriate visual and statistical methods. If the assumption of sphericity were violated, a Greenhouse–Geisser correction was employed. Potential changes in pain sensitivity (PPT, PTT, TSP, and CPM), gait (speed and stride length), and psychological (PCS, HADS anxiety, and HADS depression) parameters measured at baseline and after the sleep disruption were compared using a repeated‐measure GLM adjusted for sex and baseline PSQI scores. Multiple linear regression was used to explain variability in peak knee pain intensity (NRS 0–10) based on baseline pain sensitivity (PPT, PTT, TSP, and CPM), cognitive factors (PCS, HADS anxiety, HADS depression, and PSQI score), and biomechanical properties (speed and stride length). No indications of multicollinearity were detected with all variability inflation factors (VIF) levels below 10. Backward selection was used to isolate the best possible model from the available independent variables. Each model is presented as the initial model and the final iteration, along with the total number of iterations. The statistical analyses were performed using SPSS (IBM SPSS Statistics for Windows, version 28.0) and RStudio (v. 2024.12.1).

## 3 Results

### 3.1 Baseline Characteristics

Thirty healthy, pain‐free volunteers (18 female) participated in the study and were included for analysis. All participants had sufficient compliance with the sleep disruption protocol, confirmed by completion of the assigned task. See Table 1 for an overview of baseline metrics.

**TABLE 1:** Baseline factors presented as mean values (standard deviation).

Baseline metric	Mean (SD)
Age (years)	26.6 (4.0)
BMI (kg/m^2^)	24.6 (3.3)
PPT (kPa)	36.6 (14.7)
PTT (kPa)	78.1 (23.2)
TSP (ΔVAS)	1.3 (1.5)
CPM (ΔkPa)	9.1 (13.8)
PSQI	5.5 (2.6)
PCS	9.2 (7.5)
HADS anxiety	4.9 (3.1)
HADS depression	1.7 (2.3)
KOOS symptoms	93.3 (8.9)
KOOS pain	94.9 (7.6)
KOOS aDL	97.8 (5.4)
KOOS sport/rec	94.0 (12.8)
KOOS QoL	92.5 (13.8)

*Note:* Overview of baseline demographics and psychophysical characteristics.

Abbreviations: ADL, activity of daily living; BMI, body mass index; CPM, conditioned pain modulation; HADS, hospital anxiety and depression scale; KOOS, knee injury and osteoarthritis outcome Score; PCS, pain catastrophizing scale; PPT, pressure pain threshold; PSQI, Pittsburgh sleep quality index; PTT, pressure tolerance threshold; QoL, quality of life; Rec, recreational, TSP, temporal summation of pain.

### 3.2 Experimental Sleep Disruption

**FIGURE 2:**
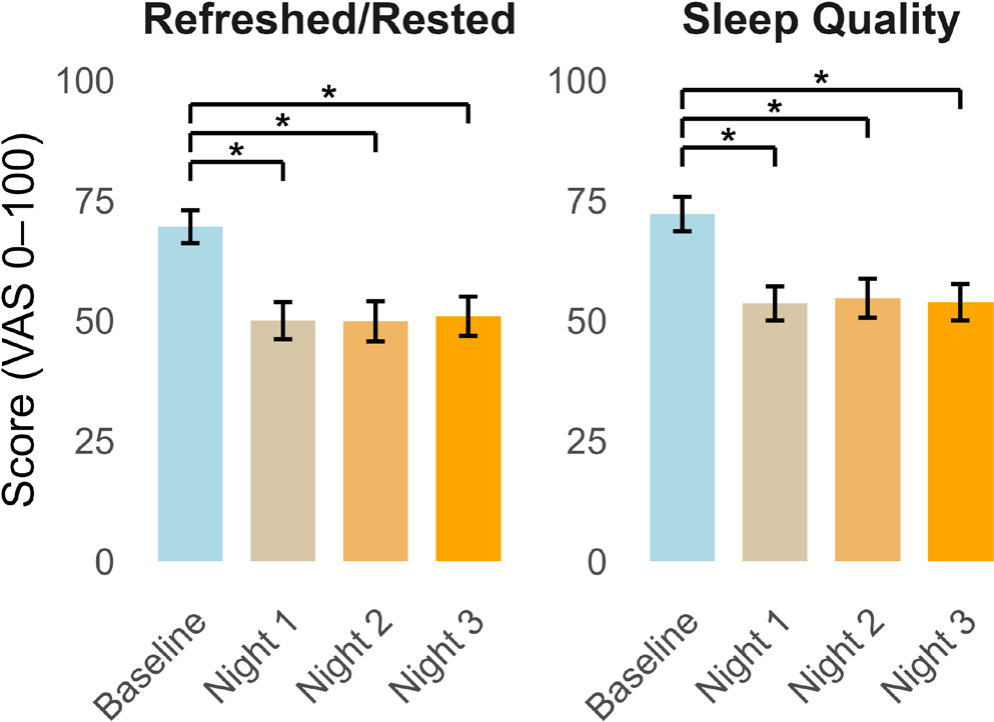
Self‐reported Level of Rest (left‐hand side) and Sleep Quality (right‐hand side) were scored from 0 (worst) to 100 (best) at baseline and after each night with experimental sleep disruption (Night 1–3). The grey ribbon represents one standard deviation. VAS, visual analogue scale. An asterisk (*) equates to *p* < 0.05.

There was a significant reduction in self‐reported quality of sleep over time (F (3.87) = 11.47, *p* < 0.01, Figure 2). Post hoc testing (Bonferroni corrected) revealed that the baseline quality of sleep was significantly better than the three experimental nights (*p* < 0.05). Similarly, there was a significant reduction in self‐reported level of rest over time (F (3.87) = 12.45, *p* < 0.001, Figure 2). Post hoc testing (Bonferroni‐corrected) revealed that the baseline level of rest was significantly better than the three experimental nights (*p* < 0.05).

Participants spent between 17.38 and 25.63 (14.89–18.33) minutes awake per night compared to an average of 2.17 min spent awake during the night at baseline. There was a significant increase in awake time during the night over time (F(1.90, 53.07) = 11.49, *p* < 0.001). Post hoc analysis (Bonferroni‐corrected) revealed that the participants spent significantly more time awake during all three experimental nights (*p* < 0.001) compared to baseline. Participants had between 2.73 and 3.17 (0.75–1.14) awakenings per night related to the forced awakenings compared to an average of 0.40 (0.72) awakenings at baseline. An overview of the self‐reported sleep metrics can be found in Table 2. There was a significant increase in the number of awakenings over time (F (3, 87) = 64.73, *p* < 0.001). Post hoc analysis (Bonferroni corrected) revealed that the participants had significantly more awakenings during all three experimental nights compared to baseline (*p* < 0.001).

**TABLE 2:** Self‐reported sleep metrics at baseline (B) and the nights with experimental sleep disruption (*N*1‐3).

SLEEP METRIC	B	*N*1	*N*2	*N*3
Bedtime (*n* before midnight)	22	19	16	20
Sleep Latency (mean (SD), minutes)	20.9 (21.8)	19.0 (13.1)	13.6 (11.5)	16.8 (22.9)
Total sleep time (mean (SD), minutes)	456.0 (78.0)	470.0 (101.6)	475.6 (71.6)	461.8 (104.5)
Awakenings (mean (SD) per night)	0.4 (0.7)	3.2 (0.7)	2.7 (1.1)	2.9 (1.1)
Awake time (mean (SD), minutes)	2.1 (4.0)	25.6 (22.8)	17.4 (14.8)	18.6 (18.3)

*Note:* Overview of sleep metrics at baseline and during the experimental nights.

Abbreviations: B, baseline; *N*, night; *n*, number; SD, standard deviation.

### 3.3 Experimental Knee Pain

**FIGURE 3:**
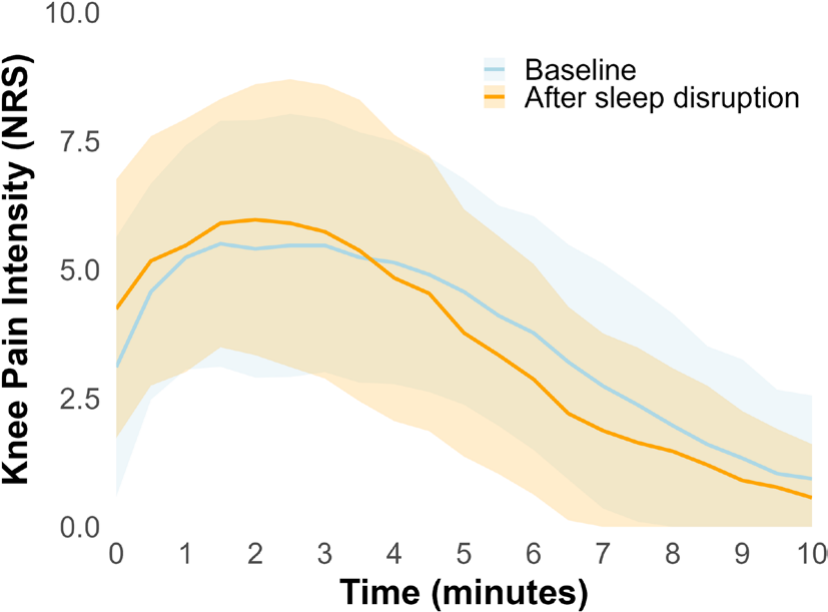
Pain development following injection with hypertonic saline (7%) in the infrapatellar fat pad. Shown for baseline (blue) and follow‐up (orange). Pain intensity was rated on an NRS every 30 s for 10 min. The blue and orange ribbons represent one standard deviation for baseline and follow‐up pain scores, respectively. NRS, numeric rating scale.

**FIGURE 4:**
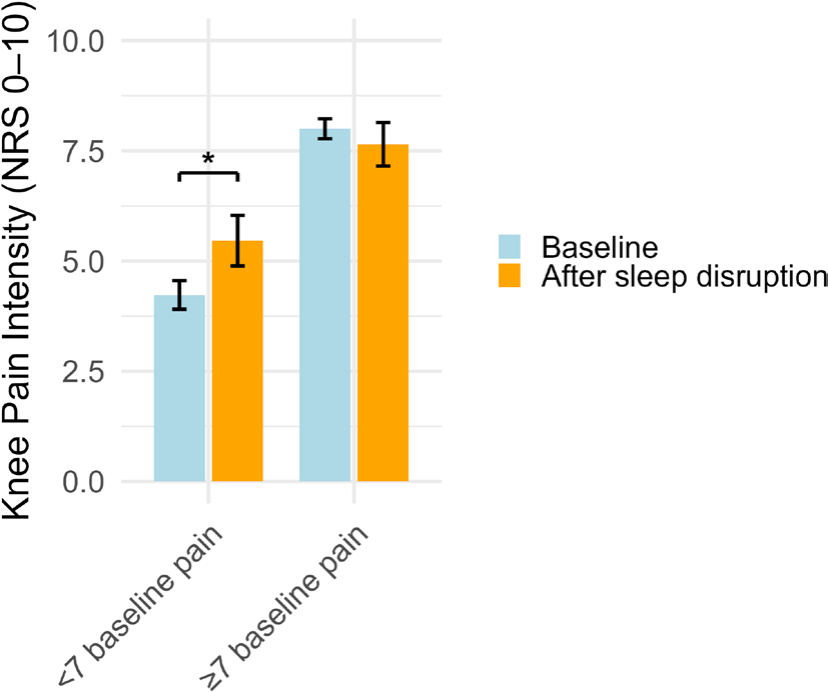
Exploratory comparison of peak knee pain at baseline and after the sleep disruption for participants who had baseline knee pain above (*n* = 17) and below (*n* = 13) *seven*. NRS, numeric rating scale. An asterisk (*) equates to p < 0.05.

The knee pain intensity peaked at VAS (0–10) 6.4 (2.2) at baseline and at 6.7 (2.3) after the sleep disruption. Knee pain intensity changed significantly over the 15 min of recording (F(2.27, 65.79) = 80.91, *p* < 0.001, Figure 3). Post hoc analysis (Bonferroni corrected) revealed that the participants had significant increases in knee pain intensity from immediately after the injection (zero seconds) to 1 min after the injection (*p* < 0.001). From 5 to 8 min after the injection, there was a significant decrease in the pain intensity every 30 s (*p* < 0.01). There were significant differences in knee pain intensity over time when comparing baseline to after the sleep disruption (F(3.28, 95.22) = 5.02, *p* < 0.01, Figure 3). Post hoc analysis (Bonferroni corrected) revealed that the knee pain intensity was higher immediately after the injection after the sleep disruption, compared to baseline (*p* < 0.05). Further, the pain was significantly lower after sleep disruption from 5 min and 30 s to 8 min after the injection compared to habitual sleep (*p* < 0.05). There was no significant effect of day on knee pain intensity Figure 4.

### 3.4 Quantitative Sensory Testing

There was no significant difference in any QST measures after the sleep disruptions compared to baseline, either unadjusted or adjusted for baseline PSQI and sex.

### 3.5 Gait Biomechanics

There were no significant differences in any biomechanical variables after the sleep disruptions compared to baseline, either unadjusted or adjusted for baseline PSQI and sex.

### 3.6 Psychological Factors

There were no significant differences in HADS anxiety, HADS depression, or PCS after the sleep disruptions compared to baseline, either unadjusted or adjusted for baseline PSQI and sex.

### 3.7 Explorative Analysis of Pain‐Severity Subgroups

An exploratory analysis was conducted to estimate the influence of baseline knee pain intensity. To do this, the participants were dichotomized into two groups based on their baseline NRS knee pain scores, with a cut‐off of seven based on the most reported cutoff for severe pain (severe ≥ 7 > less severe) (Monticelli and Van Grootven 2025). When adjusting the repeated‐measures ANOVA for severity of baseline knee pain intensity, participants with less severe baseline knee pain intensity (*n* = 13) had significantly higher pain after the sleep disruption (F(1, 28) = 6.30, *p* < 0.05).

## 4 Discussion

The current study induced experimental knee pain by injecting hypertonic saline into the infrapatellar fat pad, with an average peak knee pain intensity of around 6.5/10 and significantly reduced sleep quality and level of rest by enforcing three awakenings per night for three nights. This experimental framework was unable to provoke changes in experimental knee pain intensity, pain sensitivity, gait pattern, or psychological factors.

### 4.1 Experimental Knee Pain in Combination With Sleep Disruption

The participants in the current study had moderate to severe peak knee pain after an injection with hypertonic saline in the infrapatellar fat pad, as expected based on previous reports (Henriksen et al. 2011; Hodges et al. 2009). Similarly, the experimental sleep disruption successfully lowered both sleep quality and level of rest, also in accordance with previous reports (Joensen et al. 2025). However, pain sensitivity, gait pattern, and psychological factors measured remained unchanged after the sleep disruption in the current study. Generally, experimental sleep deprivation is indicated to produce hyperalgesic changes in healthy humans (Kundermann et al. 2004). Supporting this, previous studies using the same experimental sleep disruption (Hertel et al. 2023; Joensen et al. 2025), more extensive experimental sleep disruption (Smith et al. 2007), partial sleep restriction (Tiede et al. 2010), or total sleep deprivation (Hertel, Sathiyalingam, et al. 2024; Palsson et al. 2023; Staffe et al. 2019) have been successful in changing pain sensitivity. The hyperalgesic effects previously observed after experimental sleep disruptions have been suggested to be consequential to a reduced capacity of top‐down pain control (Tiede et al. 2010). Nevertheless, it is important to note that short‐term sleep disruption cannot be compared to chronic sleep disturbances and should not be able to provoke the effects observed in, e.g., chronic pain patients with poor sleep quality. Furthermore, a recent study found that only healthy participants with good baseline sleep quality displayed changes in pain sensitivity and psychological parameters after experimental sleep disruption (Joensen et al. 2025). In the current study, the average PSQI score is above the cut‐off for poor sleep quality (Buysse et al. 1989), which could indicate that people who already have poor sleep quality at baseline are more resilient to experimental disturbances in their sleep, conceivably because any maladaptive changes already are in effect. Finally, the pre‐determined awakenings employed in the current work do not control which sleep stage is interrupted. Generally, slow‐wave sleep (SWS) is believed to be the most restorative sleep stage (Bjurstrom and Irwin 2016) and is given the most emphasis for sleep disruptions' adverse effects on pain sensitivity (Kundermann et al. 2004), as SWS deprivation has been shown to induce hyperalgesia, increase inflammation, and impair endogenous modulation of pain (Lentz et al. 1999; Onen et al. 2001), and even increases pain sensitivity when total sleep time remains unchanged (Lentz et al. 1999).

Previously, sleep disturbances, but not short sleep durations, were found to be associated with increased inflammation in a meta‐analysis of experimental studies (Irwin et al. 2016). Since prostaglandins and IL‐6 are known to sensitize the peripheral nociceptors and enhance the processing of pain signals (Kawasaki et al. 2008), the sleep disturbance in the current study would be expected to increase pain sensitivity. This indicates that sleep disturbances must be of a certain duration or severity to induce these maladaptive effects. Supporting this, previous, more extensive experimental sleep disruptions have been shown to increase sensitivity measures of the peripheral and central pain pathways in healthy participants (Hertel et al. 2023; Hertel, Sathiyalingam, et al. 2024; Smith et al. 2007; Staffe et al. 2019). Furthermore, gait changes inherently change consequentially to knee pain. Several characteristics of gait have been associated with movement‐evoked pain (Boyer and Hafer 2019) and potentially variability in knee OA pain (Hutchison et al. 2022). Supporting this, the adopted gait pattern of patients with knee OA changes with pain relief, increasing its similarity to gait patterns of pain‐free individuals (Henriksen et al. 2006). In the current study, speed, stride length, peak knee flexion, and knee symmetry were not changed with experimental knee pain and/or sleep disruptions. This might indicate that longer exposure to knee pain or structural changes is required for the gait changes observed in patients with knee OA.

Previous studies have also combined sleep provocations with experimental pain models, predominantly delayed‐onset muscle soreness (DOMS) and total sleep deprivation, and demonstrated increased pain sensitivity (Hertel, Sathiyalingam, et al. 2024) and amplified DOMS (Palsson et al. 2023). These findings contrast with the current work, which might be attributed to the different sleep provocations and pain models used. Hypertonic saline provokes osmotic stress, activating chemical and mechanical afferents along with a mild local inflammatory response (Mense 1993). DOMS offers a longer duration and inflammatory exposure. Thus, hypertonic saline injections at a small site, such as the IFP, might not synergize well with sleep disturbances compared to DOMS (Newham et al. 1983; Peake et al. 2017). Alternatively, hypertonic saline provokes much higher pain intensities compared to DOMS models (Bennell et al. 2004; Hertel, Sathiyalingam, et al. 2024), and this high‐intensity pain could potentially dominate the response. Tentatively supporting this, exploratory analyses revealed that participants perceiving the baseline injection as severely painful showed no change following the sleep disruption, whereas those with milder baseline pain did. This could be a preliminary suggestion that individuals with high baseline sensitivity might have limited capacity for experimental modulation and that high‐intensity pain might dominate the response. However, the sub‐groups in this exploratory analysis are small, and this finding should be regarded as strictly hypothesis‐generating and should be tested in a larger sample.

### 4.2 Methodological Considerations

The sleep disruption protocol used in the current study has previously been demonstrated to increase pain sensitivity in healthy individuals (Joensen et al. 2025), and the number of awakenings per night is chosen to reflect clinical reports from patients with chronic pain (Morin et al. 1998). Nevertheless, patients with chronic pain experience these disturbances over long periods of time, while the duration in this experiment was only three nights. Future studies should prolong the period with awakenings to better mimic the clinical reality or adopt more extensive sleep disturbance protocols, such as total sleep deprivation. Further, a limitation of the present design is the lack of control conditions for both the sleep disruption and experimental knee pain. While this prevents separation of main and interaction effects, repeated hypertonic‐saline injections have previously been demonstrated to show no meaningful habituation (Smith et al. 2023), reducing the likelihood that repetition should influence the results. Finally, this model is novel, and therefore, the sample size calculation was based on an estimation of the effect size. Following the initiation of data collection for the current study, Joensen et al. (2025) demonstrated changes in pain intensity following a 120% PPT stimulation after three nights of sleep disruption. The effect size was 0.34 (Cohen's d), which yields an estimated sample size of 70 subjects. However, 120% PPT and hypertonic saline injections differ in modality, duration, and intensity, and the hypertonic saline injection was expected to produce pain of a longer duration and higher intensity. Nevertheless, the sample size of the current study may be slightly underpowered.

## 5 Conclusion

The current study introduced a novel model combining experimental knee pain and repeated sleep disruptions to better mimic the features of knee osteoarthritis by increasing the complexity of the model. The hypertonic saline elicited knee pain, and participants reported reduced sleep quality after the experimental sleep disruptions. No changes were found in measures of pain severity or psychological parameters. These findings suggest that short‐term sleep disturbances alone might be insufficient to change pain processing. Future studies should attempt to disentangle the effects of experimental sleep disruption and knee pain by adding a control condition to both modalities.

## Author Contributions

Funding was secured by Kristian Kjær‐Staal Petersen, Michael Skipper Andersen, and Lars Arendt‐Nielsen. The study was conceptualized by Emma Hertel, Aske Hostrup Graversen, Anders Bobo Larsen, and Kristian Kjær‐Staal Petersen. Emma Hertel, Aske Hostrup Graversen, and Anders Bobo Larsen conducted the experiment. Emma Hertel performed the data analysis and visualization, while Diego Martinez Echevarria computed the biomechanical variables. All authors critically reviewed the results. Emma Hertel drafted the manuscript, which was edited by Kristian Kjær‐Staal Petersen. All authors reviewed and approved the final version of the manuscript and agree to be accountable for all aspects of the work.

## Funding

Center for Neuroplasticity and Pain (CNAP) is supported by the Danish National Research Foundation (DNRF121). The Center for Mathematical Modelling of Knee Osteoarthritis (MathKOA) is funded by the Novo Nordisk Foundation (NNF21OC0065373).

## Conflicts of Interest

The authors declare no conflicts of interest.

## References

[ejp70260-bib-0001] Bennell, K. , P. Hodges , R. Mellor , C. Bexander , and T. Souvlis . 2004. “The Nature of Anterior Knee Pain Following Injection of Hypertonic Saline Into the Infrapatellar Fat Pad.” Journal of Orthopaedic Research 22: 116–121. 14656669 10.1016/S0736-0266(03)00162-1

[ejp70260-bib-0002] Bjelland, I. , A. A. Dahl , T. T. Haug , and D. Neckelmann . 2002. “The Validity of the Hospital Anxiety and Depression Scale: An Updated Literature Review.” Journal of Psychosomatic Research 52: 69–77. 11832252 10.1016/s0022-3999(01)00296-3

[ejp70260-bib-0003] Bjurstrom, M. F. , and M. R. Irwin . 2016. “Polysomnographic Characteristics in Nonmalignant Chronic Pain Populations: A Review of Controlled Studies.” Sleep Medicine Reviews 26: 74–86. 10.1016/j.smrv.2015.03.004 PMC4598249 26140866

[ejp70260-bib-0004] Boyer, K. A. , and J. F. Hafer . 2019. “Gait Mechanics Contribute to Exercise Induced Pain Flares in Knee Osteoarthritis.” BMC Musculoskeletal Disorders 20: 1–10. 30871519 10.1186/s12891-019-2493-4 PMC6419357

[ejp70260-bib-0005] Buysse, D. J. , C. F. Reynolds , T. H. Monk , S. R. Berman , and D. J. Kupfer . 1989. “The Pittsburgh Sleep Quality Index: A New Instrument for Psychiatric Practice and Research.” Psychiatry Research 28: 193–213. 2748771 10.1016/0165-1781(89)90047-4

[ejp70260-bib-0006] Collins, N. J. , C. A. C. Prinsen , R. Christensen , E. M. Bartels , C. B. Terwee , and E. M. Roos . 2016. “Knee Injury and Osteoarthritis Outcome Score (KOOS): Systematic Review and Meta‐Analysis of Measurement Properties.” Osteoarthritis and Cartilage 24: 1317–1329. 27012756 10.1016/j.joca.2016.03.010

[ejp70260-bib-0007] Enqvist, J. , L. Joakim Holmberg , M. S. Andersen , and A. Arndt . 2025. “Ground Reaction Force Asymmetry Underestimates Asymmetries in Knee Joint Reaction Forces During Countermovement Jumps.” Journal of Biomechanics 189: 112834. 40592095 10.1016/j.jbiomech.2025.112834

[ejp70260-bib-0008] Felson, D. T. 2005. “The Sources of Pain in Knee Osteoarthritis.” Current Opinion in Rheumatology 17: 624–628. 16093843 10.1097/01.bor.0000172800.49120.97

[ejp70260-bib-0009] Finan, P. H. , B. R. Goodin , and M. T. Smith . 2013. “The Association of Sleep and Pain: An Update and a Path Forward.” Journal of Pain 14: 1539–1552. 24290442 10.1016/j.jpain.2013.08.007 PMC4046588

[ejp70260-bib-0010] Graven‐Nielsen, T. , M. Izumi , K. K. Petersen , and L. Arendt‐Nielsen . 2017. “User‐Independent Assessment of Conditioning Pain Modulation by Cuff Pressure Algometry.” European Journal of Pain 21: 552–561. 27859944 10.1002/ejp.958

[ejp70260-bib-0011] Graven‐Nielsen, T. , H. B. Vaegter , S. Finocchietti , G. Handberg , and L. Arendt‐Nielsen . 2015. “Assessment of Musculoskeletal Pain Sensitivity and Temporal Summation by Cuff Pressure Algometry: A Reliability Study.” Pain 156: 2193–2202. 26172551 10.1097/j.pain.0000000000000294

[ejp70260-bib-0012] Haack, M. , N. Simpson , N. Sethna , S. Kaur , and J. Mullington . 2020. “Sleep Deficiency and Chronic Pain: Potential Underlying Mechanisms and Clinical Implications.” Neuropsychopharmacology (New York, N.Y.) 45: 205–216. 10.1038/s41386-019-0439-z PMC6879497 31207606

[ejp70260-bib-0013] Henriksen, M. , S. Rosager , J. Aaboe , T. Graven‐Nielsen , and H. Bliddal . 2011. “Experimental Knee Pain Reduces Muscle Strength.” Journal of Pain 12: 460–467. 21146464 10.1016/j.jpain.2010.10.004

[ejp70260-bib-0014] Henriksen, M. , E. B. Simonsen , T. Alkjær , et al. 2006. “Increased Joint Loads During Walking – A Consequence of Pain Relief in Knee Osteoarthritis.” Knee 13: 445–450. 17011194 10.1016/j.knee.2006.08.005

[ejp70260-bib-0015] Hertel, E. , L. Arendt‐Nielsen , A. E. Olesen , M. S. Andersen , and K. K.‐S. Petersen . 2024. “Quantitative Sensory Testing, Psychological Factors, and Quality of Life as Predictors of Current and Future Pain in Patients With Knee Osteoarthritis.” Pain 165: 1719–1726. 38381930 10.1097/j.pain.0000000000003194

[ejp70260-bib-0016] Hertel, E. , M. E. McPhee , and K. K. K. Petersen . 2023. Investigation of pain sensitivity following 3 nights of disrupted sleep in healthy individuals. 10.1002/ejp.2101 36862019

[ejp70260-bib-0017] Hertel, E. , E. Sathiyalingam , L. Pilgaard , S. J. Brommann , R. Giordano , and K. K. S. Petersen . 2024. “Psychophysical Changes After Total Sleep Deprivation and Experimental Muscle Pain.” Journal of Sleep Research 34, no. 2: e14329. 39289848 10.1111/jsr.14329 PMC11911060

[ejp70260-bib-0018] Hodges, P. W. , R. Mellor , K. Crossley , and K. Bennell . 2009. “Pain Induced by Injection of Hypertonic Saline Into the Infrapatellar Fat Pad and Effect on Coordination of the Quadriceps Muscles.” Arthritis Care & Research (Hoboken) 61: 70–77. 10.1002/art.24089 19116977

[ejp70260-bib-0019] Huber, F. A. , T. A. Toledo , G. Newsom , and J. L. Rhudy . 2022. “The Relationship Between Sleep Quality and Emotional Modulation of Spinal, Supraspinal, and Perceptual Measures of Pain.” Biological Psychology 171: 108352. 35569574 10.1016/j.biopsycho.2022.108352

[ejp70260-bib-0020] Hutchison, L. , J. Grayson , C. Hiller , N. D'Souza , S. Kobayashi , and M. Simic . 2022. “Relationship Between Knee Biomechanics and Pain in People With Knee Osteoarthritis: A Systematic Review and Meta‐Analysis.” Arthritis Care & Research (Hoboken) 75: 1351–1361. 10.1002/acr.25001 35997473

[ejp70260-bib-0021] Imai, Y. , K. K. Petersen , C. D. Mørch , and L. Arendt Nielsen . 2016. “Comparing Test–Retest Reliability and Magnitude of Conditioned Pain Modulation Using Different Combinations of Test and Conditioning Stimuli.” 10.1080/08990220.2016.1229178 27650216

[ejp70260-bib-0022] Irwin, M. R. , R. Olmstead , and J. E. Carroll . 2016. “Sleep Disturbance, Sleep Duration, and Inflammation: A Systematic Review and Meta‐Analysis of Cohort Studies and Experimental Sleep Deprivation.” Biological Psychiatry 80: 40–52. 26140821 10.1016/j.biopsych.2015.05.014 PMC4666828

[ejp70260-bib-0023] Joensen, E. D. R. , L. Frederiksen , S. Vindbaek Frederiksen , et al. 2025. “Sex and Sleep Quality Effects on the Relationship Between Sleep Disruption and Pain Sensitivity.” European Journal of Pain 29: e70023. 40197999 10.1002/ejp.70023 PMC11977682

[ejp70260-bib-0024] Kasović, M. , A. Štefan , and L. Štefan . 2021. “The Associations Between Objectively Measured Gait Speed and Subjective Sleep Quality in First‐Year University Students, According to Gender.” Nature and Science of Sleep 13: 1663–1668. 10.2147/NSS.S328218 PMC8478338 34594142

[ejp70260-bib-0025] Kawasaki, Y. , L. Zhang , J.‐K. Cheng , and R.‐R. Ji . 2008. “Cytokine Mechanisms of Central Sensitization: Distinct and Overlapping Role of Interleukin‐1β, Interleukin‐6, and Tumor Necrosis Factor‐α in Regulating Synaptic and Neuronal Activity in the Superficial Spinal Cord.” Journal of Neuroscience 28: 5189–5194. 18480275 10.1523/JNEUROSCI.3338-07.2008 PMC2408767

[ejp70260-bib-0026] Kellgren, J. H. , and J. S. Lawrence . 1957. “Radiological Assessment of Osteo‐Arthrosis.” Annals of the Rheumatic Diseases 16: 494–502. 13498604 10.1136/ard.16.4.494 PMC1006995

[ejp70260-bib-0027] Kundermann, B. , J. C. Krieg , W. Schreiber , and S. Lautenbacher . 2004. “The Effect of Sleep Deprivation on Pain.” Pain Research and Management 9: 25–32. 15007400 10.1155/2004/949187

[ejp70260-bib-0028] Lentz, M. , C. Landis , J. Rothermel , and J. Shaver . 1999. “Effects of Selective Slow Wave Sleep Disruption on Musculoskeletal Pain and Fatigue in Middle Aged Women.” Journal of Rheumatology 26, no. 7: 1586–1592. 10405949

[ejp70260-bib-0029] Li, L. , Z. Li , Y. Li , X. Hu , Y. Zhang , and P. Fan . 2020. “Profiling of Inflammatory Mediators in the Synovial Fluid Related to Pain in Knee Osteoarthritis.” BMC Musculoskeletal Disorders 21: 1–10. 10.1186/s12891-020-3120-0 PMC7023718 32059658

[ejp70260-bib-0030] Maly, M. R. , P. A. Costigan , and S. J. Olney . 2008. “Mechanical Factors Relate to Pain in Knee Osteoarthritis.” Clinical biomechanics 23: 796–805. 18346827 10.1016/j.clinbiomech.2008.01.014

[ejp70260-bib-0031] McPhee, M. , and T. Graven‐Nielsen . 2019. “Alterations in Temporal Summation of Pain and Conditioned Pain Modulation Across an Episode of Experimental Exercise‐Induced Low Back Pain.” Journal of Pain 20: 264–276. 30236748 10.1016/j.jpain.2018.08.010

[ejp70260-bib-0032] Medic, G. , M. Wille , and M. E. H. Hemels . 2017. “Short‐ and Long‐Term Health Consequences of Sleep Disruption.” Nature and Science of Sleep 9: 151–161. 10.2147/NSS.S134864 PMC5449130 28579842

[ejp70260-bib-0033] Mense, S. 1993. “Nociception From Skeletal Muscle in Relation to Clinical Muscle Pain.” Pain 54: 241–289. 8233542 10.1016/0304-3959(93)90027-M

[ejp70260-bib-0034] Monticelli, A. , and B. Van Grootven . 2025. “Exploring Established Cut‐Off Points for Pain Levels in the Numeric Rating Scale: Insights From a Literature Overview.” Pain Management Nursing 26: 689–695. 40915874 10.1016/j.pmn.2025.08.005

[ejp70260-bib-0035] Mora, J. C. , R. Przkora , and Y. Cruz‐Almeida . 2018. “Knee Osteoarthritis: Pathophysiology and Current Treatment Modalities.” Journal of Pain Research 11: 2189. 30323653 10.2147/JPR.S154002 PMC6179584

[ejp70260-bib-0036] Morin, C. M. , D. Gibson , and J. Wade . 1998. Self‐reported sleep and mood disturbance in chronic pain patients. 14. 10.1097/00002508-199812000-00007 9874009

[ejp70260-bib-0037] Neogi, T. 2013. “The Epidemiology and Impact of Pain in Osteoarthritis.” Osteoarthritis and Cartilage 21: 1145–1153. 10.1016/j.joca.2013.03.018 PMC3753584 23973124

[ejp70260-bib-0038] Newham, D. J. , G. McPhail , K. R. Mills , and R. H. T. Edwards . 1983. “Ultrastructural Changes After Concentric and Eccentric Contractions of Human Muscle.” Journal of the Neurological Sciences 61: 109–122. 6631446 10.1016/0022-510x(83)90058-8

[ejp70260-bib-0039] Onen, S. H. , A. Alloui , A. Gross , A. Eschallier , and C. Dubray . 2001. “The Effects of Total Sleep Deprivation, Selective Sleep Interruption and Sleep Recovery on Pain Tolerance Thresholds in Healthy Subjects.” Journal of Sleep Research 10: 35–42. 11285053 10.1046/j.1365-2869.2001.00240.x

[ejp70260-bib-0040] Palsson, T. S. , A. Rubio‐Peirotén , and V. Doménech‐García . 2023. “Sleep Deprivation Increases Pain Sensitivity Following Acute Muscle Soreness.” Sleep Medicine 109: 75–81. 37423022 10.1016/j.sleep.2023.06.010

[ejp70260-bib-0041] Peake, J. M. , O. Neubauer , P. A. D. Gatta , and K. Nosaka . 2017. “Muscle Damage and Inflammation During Recovery From Exercise.” Journal of Applied Physiology (1985) 122: 559–570. 10.1152/japplphysiol.00971.2016 28035017

[ejp70260-bib-0042] Petersen, K. K. , O. Simonsen , A. E. Olesen , C. D. Mørch , and L. Arendt‐Nielsen . 2019. “Pain Inhibitory Mechanisms and Response to Weak Analgesics in Patients With Knee Osteoarthritis.” European Journal of Pain 23: 1904–1912. 31376308 10.1002/ejp.1465

[ejp70260-bib-0043] Petersen, K. K. K. K. , T. Graven‐Nielsen , O. Simonsen , M. B. M. B. Laursen , and L. Arendt‐Nielsen . 2016. “Preoperative Pain Mechanisms Assessed by Cuff Algometry Are Associated With Chronic Postoperative Pain Relief After Total Knee Replacement.” Pain 157: 1400–1406. 27331347 10.1097/j.pain.0000000000000531

[ejp70260-bib-0044] Petersen, K. K. S. , K. Kilic , E. Hertel , et al. 2023. “Quantitative Sensory Testing as an Assessment Tool to Predict the Response to Standard Pain Treatment in Knee Osteoarthritis: A Systematic Review and Meta‐Analysis.” Pain Rep 8: e1079. 38699564 10.1097/PR9.0000000000001079 PMC11065125

[ejp70260-bib-0045] Smith, M. T. , R. R. Edwards , U. D. McCann , and J. A. Haythornthwaite . 2007. “The Effects of Sleep Deprivation on Pain Inhibition and Spontaneous Pain in Women.” Sleep 30: 494–505. 17520794 10.1093/sleep/30.4.494

[ejp70260-bib-0046] Smith, S. A. , R. Norbury , A. J. Hunt , and A. R. Mauger . 2023. “Intra‐ and Interindividual Reliability of Muscle Pain Induced by an Intramuscular Injection of Hypertonic Saline Injection Into the Quadriceps.” European Journal of Pain 27: 1216–1225. 37376739 10.1002/ejp.2151

[ejp70260-bib-0047] Staffe, A. T. , M. W. Bech , S. L. K. Clemmensen , H. T. Nielsen , D. B. Larsen , and K. K. Petersen . 2019. “Total Sleep Deprivation Increases Pain Sensitivity, Impairs Conditioned Pain Modulation and Facilitates Temporal Summation of Pain in Healthy Participants.” PLoS One 14: e0225849. 31800612 10.1371/journal.pone.0225849 PMC6892491

[ejp70260-bib-0048] Sullivan, M. J. L. , S. R. Bishop , and J. Pivik . 1995. “The Pain Catastrophizing Scale: Development and Validation.” Psychological Assessment 7: 524–532.

[ejp70260-bib-0049] Sun, Y. , I. Laksono , J. Selvanathan , et al. 2021. “Prevalence of Sleep Disturbances in Patients With Chronic Non‐Cancer Pain: A Systematic Review and Meta‐Analysis.” Sleep Medicine Reviews 57: 101467. 33827029 10.1016/j.smrv.2021.101467

[ejp70260-bib-0050] Svetek, A. , K. Morgan , J. Burland , and N. R. Glaviano . 2025. “Validation of OpenCap on Lower Extremity Kinematics During Functional Tasks.” Journal of Biomechanics 183: 112602. 40048968 10.1016/j.jbiomech.2025.112602

[ejp70260-bib-0051] Tiede, W. , W. Magerl , U. Baumgärtner , B. Durrer , U. Ehlert , and R. D. Treede . 2010. “Sleep Restriction Attenuates Amplitudes and Attentional Modulation of Pain‐Related Evoked Potentials, but Augments Pain Ratings in Healthy Volunteers.” Pain 148: 36–42. 19864066 10.1016/j.pain.2009.08.029

[ejp70260-bib-0052] Umemura, G. S. , J. P. Pinho , J. Duysens , H. I. Krebs , and A. Forner‐Cordero . 2021. “Sleep deprivation affects gait control.” Scientific Reports 11: 1–11. 34702960 10.1038/s41598-021-00705-9 PMC8548553

[ejp70260-bib-0053] Wiklund, T. , B. Gerdle , S. J. Linton , E. Dragioti , and B. Larsson . 2020. “Insomnia Is a Risk Factor for Spreading of Chronic Pain: A Swedish Longitudinal Population Study (SwePain).” European Journal of Pain 24: 1348–1356. 32386443 10.1002/ejp.1582

